# White paper on effective preacquisition evaluation of soft tissue robotic surgery platforms for healthcare institutions

**DOI:** 10.1007/s00464-026-12637-4

**Published:** 2026-02-24

**Authors:** Ankit Sarin, Dimitrios Stefanidis, Jay A. Redan, Paresh C. Shah, Patricia Sylla

**Affiliations:** 1 Davis Health, University of California, Sacramento, CA USA; 2 Indiana University School of Medicine, Indianapolis, IN USA; 3 Advent Health-Celebration, University of Central Florida College of Medicine, Celebration, FL USA; 4 Weill Cornell Medical Center, New York Presbyterian, New York, NY USA; 5 Mount Sinai Health System, New York, NY USA

**Keywords:** Robotic surgery, Selection framework, Healthcare economics, Multi-criteria decision analysis, IDEAL framework, Surgical robotics, Surgical education

## Abstract

**Background:**

With the increasing popularity of robotic surgery among surgeons, the selection of soft tissue robotic surgery platforms represents a critical strategic decision for healthcare institutions. Following patent expirations in robotic technology, institutions face increasingly complex choices among diverse platforms with varying capabilities, costs, and implementation requirements. The goal of this white paper was to define a framework for evaluating robotic surgical platforms to aid acquisition decisions by healthcare institutions.

**Methods:**

Based on expert robotic surgeon opinion informed by the IDEAL Framework (Idea, Development, Exploration, Assessment, and Long-term study) and Multi-Criteria Decision Analysis (MCDA) methodology, and discussions with industry stakeholders, core decision domains for the acquisition of a robotic system were defined and a comprehensive framework for evaluating robotic surgical platforms was developed. The impact of procedure, specialty, and institution-specific variations of the selection criteria was explored.

**Results:**

Eight core decision domains were identified: clinical outcomes and patient relevance, hospital-specific considerations, technology-specific attributes, physician needs and preferences, surgeon and trainee education, economic implications, policy and regulatory factors, and long-term sustainability. According to this framework, financial sustainability timelines vary substantially based on case mix, payer composition, and implementation approach, with specialty-focused programs typically achieving positive returns more rapidly than broad multi-specialty implementations. Economic models illustrate different return-on-investment scenarios for academic medical centers, community hospitals, and specialty surgical centers. Educational infrastructure significantly impacts long-term program success, with platforms offering comprehensive simulation, dual-console training, and proficiency-based progression frameworks promoting enhanced adoption and utilization rates.

**Conclusion:**

The proposed framework provides institutions with a systematic approach to robotic platform selection designed to maximize clinical outcomes, operational efficiency, educational effectiveness, and financial sustainability, while avoiding common implementation pitfalls that have historically undermined robotic program success.

**Supplementary Information:**

The online version contains supplementary material available at 10.1007/s00464-026-12637-4.

Robotic surgery has demonstrated enhanced surgeon ergonomics, improved visualization through three-dimensional imaging systems, and increased dexterity through articulated instruments with seven degrees of freedom. [1–3] These benefits include reduced estimated blood loss, lower conversion rates to open surgery, decreased postoperative pain, and shorter hospital stays, though the magnitude of benefit varies by procedure and patient population. [4–6] However, robotic surgery presents challenges including significantly longer operative times (mean differences typically 15–45 min compared to laparoscopic approaches, varying by procedure complexity) and higher total costs compared to conventional laparoscopic approaches [5, 7, 8].

Following patent expirations in robotic technology, particularly the expiration of Intuitive Surgical’s foundational da Vinci patents beginning in 2016, [9, 10] institutions face increasingly complex choices. These choices involve diverse platforms with varying capabilities, costs, and implementation requirements. The expiration of patents has accelerated platform diversity, creating new choices among multi-port, single-port, and “laparoscopic assist” systems for soft tissue surgery, each with varying capabilities, costs, and learning curves [11].

Despite rapid adoption across numerous surgical specialties, [12] healthcare organizations lack a standardized framework for selecting appropriate robotic systems aligned with their specific needs, case mix, and strategic objectives. [13] The need for such a framework has become increasingly urgent as the robotic surgery marketplace expands beyond the traditional monopoly, with multiple new platforms entering the market, each offering different value propositions, pricing models, and technological approaches. Acquisition costs range from $500,000 to $2.5 million per system depending on platform generation and configuration. [14] The latest da Vinci 5 system is priced approximately 30% higher than previous generation systems. [15, 16] Additionally, annual service contracts exceed $100,000, with maintenance costs representing 8–12% of initial acquisition costs annually. [14, 17] Consequently, institutional commitment to robotic technology represents a major capital investment with long-term operational implications.

This white paper presents a decision-making framework applicable across various healthcare settings, incorporating established evaluation methodologies like the IDEAL Framework [18] while accounting for comprehensive institutional needs. We present specific recommendations for the selection of soft tissue robotic platforms and provide flowcharts and checklists that will aid institutions in their decision-making. 

## Methods

This framework was developed through a multi-faceted approach combining literature review, expert opinion, and established evaluation methodologies. The authors, representing expertise in robotic surgery, surgical education, and healthcare technology assessment, conducted a review of available literature on robotic platform evaluation and selection. This was supplemented by discussions with industry stakeholders and analysis of institutional experiences with robotic technology implementation.

The framework development was informed by two established methodologies:

The IDEAL Framework (Idea, Development, Exploration, Assessment, and Long-term study) provides structure for evaluating surgical innovations. [18] This approach categorizes technologies through progressive stages: Stage 0 (pre-clinical), Stage 1 (first-in-human), Stage 2a (prospective development), Stage 2b (early assessment), Stage 3 (assessment through comparative studies), and Stage 4 (long-term surveillance). Most current robotic platforms remain in early IDEAL stages (0–2b), [19] with only established systems reaching Stages 3–4. [20] This classification system helped identify the maturity level of different robotic platforms and their associated evidence base.

Multi-Criteria Decision Analysis (MCDA) methodology was employed to systematically identify and organize key decision domains influencing surgical technology adoption: Clinical Outcomes, Patient Relevance, Hospital-Specific Factors, Technology Attributes, Physician Needs, Economic Implications, Policy/Regulatory Considerations, and Sustainability. [13] This structured approach enables institutions to assign weighted importance to various evaluation criteria based on their specific contexts and priorities. [21] International consensus expert panels emphasize standardized assessment methodologies that consider technical performance, workforce training requirements, economic sustainability, and integration with existing clinical workflows. [22] 

Based on these methodologies and expert consensus, eight core decision domains were defined for robotic platform evaluation. The framework was then examined for applicability across different procedural, specialty, and institutional contexts to ensure broad utility. Economic modeling was performed using published data and institutional experiences to project return-on-investment scenarios for different healthcare settings.

## Results

Through the application of the IDEAL Framework and MCDA methodology, we identified eight critical domains that institutions must evaluate when selecting robotic surgical platforms. These domains encompass clinical, operational, technological, educational, economic, regulatory, and sustainability considerations. The framework’s application revealed significant variations in platform selection criteria based on institutional type, specialty focus, and procedural complexity.

### Eight critical domains for comprehensive platform assessment

1. *Clinical outcomes and patient relevance*: Centers on evidence-based evaluation of platform performance for specific procedures using established assessment frameworks. Key metrics include safety profiles, complication rates, operative outcomes, and patient-reported quality measures that directly impact clinical decision-making with established frameworks for measuring perioperative morbidity, mortality, and functional outcomes across surgical specialties [23, 24].
2. *Hospital-specific considerations*: Addresses operational integration challenges including infrastructure compatibility, workflow disruption, and strategic alignment. Critical factors include managing multi-vendor environments, complexity of added inventory, standardizing staff training across platforms, and optimizing case scheduling. Successful programs report that pre-implementation planning significantly reduces operational disruptions [25].
3. *Technology-Specific Attributes*: Encompasses both hardware capabilities (instrument articulation, visualization quality, haptic feedback) and digital ecosystem features (data analytics, AI integration, operational metrics dashboards, fleet management tools). Institutions must evaluate whether integrated ecosystems or modular approaches better serve their needs.
4. *Physician needs and preferences*: Captures end-user requirements through systematic assessment of ergonomics, learning curves, and interface design. Surgeon buy-in directly correlates with program utilization and success.
5. *Surgeon and trainee education*: Evaluate educational infrastructure, including simulation fidelity, proficiency-based progression, and curriculum integration. The educational ecosystem should support both initial skills acquisition and ongoing professional development while ensuring trainee progression without compromising patient safety [26] Platforms must support skills acquisition while maintaining patient safety.
6. *Economic implications*: Requires comprehensive financial modeling beyond acquisition costs to include operational expenses, reimbursement landscapes, and ROI projections. Five-year total cost of ownership models should reflect institution-specific payer mix and case volumes [27].
7. *Policy, regulatory, and ethical factors*: Ensures compliance with evolving regulations including credentialing standards, data privacy laws, and cybersecurity requirements. Institutions must carefully evaluate liability management when platforms involve vendor data exchange or cloud-based analytics [28].
8. *Long-term sustainability*: Assesses vendor stability, technology roadmaps, and environmental impact. Considerations include upgrade pathways, compatibility with emerging technologies like AI, and alignment with institutional sustainability goals as regulatory focus on healthcare’s environmental footprint intensifies. [29, 30] Environmental impact considerations, including carbon footprint, energy consumption, number of disposables, and recycling options, are increasingly important as well.

These eight domains form an interconnected framework where decisions in one area significantly impact others. Institutions must weigh these domains according to their specific priorities, with academic centers potentially emphasizing educational infrastructure, while community hospitals may prioritize economic sustainability. The following sections detail how these domains apply across different institutional settings and surgical specialties.

### Institution-specific considerations:

The application of this framework varies significantly across healthcare settings, requiring tailored approaches for different institutional types.

#### Academic hospitals

These institutions face unique challenges related to high case volumes, multi-specialty practices, with a higher proportion of inpatient cases, more urgent/emergent procedures, and greater case complexity. Access to robotic systems, case prioritization, and staffing pose distinctive challenges for academic centers. These institutions typically require dual-console operation for education, comprehensive data collection for performance improvement initiatives, and advanced systems for complex procedures. Specialized credentialing pathways must exceed minimum standards to ensure trainee proficiency without compromising patient safety. [28] Academic centers face unique economic challenges due to their payer mix and case complexity, requiring higher case volumes for financial sustainability. [25, 31] (see Financial Models section for detailed analysis).

#### Community hospitals

These facilities prioritize cost-effectiveness, versatility, and efficient resource utilization. Their higher proportion of outpatient procedures creates more opportunity for streamlining and efficient allocation of robotic systems. Selection should favor platforms with broad applications across specialties, streamlined setup for high-turnover environments, and favorable economics for mid-volume programs. Successful implementation typically begins with procedures offering established benefits and favorable reimbursement before expanding. These institutions often benefit from platforms with smaller footprints and lower per-case costs that align with their reimbursement profiles [12].

#### Surgery centers

Focus on specialized, high-volume procedures with emphasis on efficiency and patient satisfaction. These facilities benefit from compact platforms with rapid deployment capabilities and low per-case costs. Specialized centers performing focused procedures often achieve profitability within 12–18 months at lower case volumes (see Financial Models for specific thresholds). Their success stems from optimized workflows, consistent case types, and high commercial payer mix. Platform selection should prioritize reliability, ease of use, and minimal infrastructure requirements [27].

Each institutional type requires different weighting of the eight evaluation domains, with academic centers emphasizing education and research capabilities, community hospitals balancing versatility with economics, and surgery centers optimizing efficiency and ROI.

### Specialty and procedure-specific considerations for platform selection

The selection process must account for distinct requirements across surgical specialties. Table 1 provides detailed criteria and priority levels for each specialty.

**Table 1:** Specialty-specific priority criteria for robotic platform selection

Specialty	Feature	Priority	Specific Evaluation Criteria
Urology	Visualization	Critical	• Near-infrared fluorescence imaging for partial nephrectomy • Stereoscopic vision quality for nerve identification • Magnification capabilities for urethral reconstruction
	Instrument dexterity	Critical	• Precision for neurovascular bundle dissection • Needle handling for urethrovesical anastomosis • Tissue manipulation in confined pelvic space
	Multi-arm capabilities	High	• Independent fourth arm control for retraction • Camera stability during complex reconstruction • Instrument triangulation in deep pelvis
	Energy devices	High	• Vessel sealing reliability for renal hilar control • Precision for nerve-sparing dissection • Smoke evacuation effectiveness
	Console ergonomics	High	• Comfort during extended pelvic cases (3 + hours) • Hand controller precision for microsurgical tasks • Foot pedal arrangement and responsiveness
General surgery	Multi-quadrant access	Critical	• Ability to operate in multiple abdominal quadrants • Port placement flexibility for varied procedures • Reach from pelvis to upper abdomen without repositioning
	Energy device integration	Critical	• Vessel sealing for mesenteric dissection • Dissection capabilities for complex pathology • Integration with established energy platforms
	Instrument variety	High	• Range of graspers for tissue manipulation • Specialized instruments for bowel handling • Retraction capabilities for hepatobiliary exposure
	Learning curve	Medium	• Intuitiveness for varied surgical team • Ease of transition from laparoscopy • Standardized setup across different procedures
	Cost per case	High	• Instrument cost for common procedures • Reusability of components • Specialized instrument requirements
Gynecology	Suturing capabilities	Critical	• Precision for fertility-sparing myomectomy • Needle handling for delicate tissue • Knot tying efficiency in confined spaces
	Visualization	High	• Discrimination of tissue planes in endometriosis • Visualization of ureter and vascular structures • Field of view for comprehensive staging
	Size and footprint	Medium–High	• Compatibility with outpatient surgical settings • Mobility between rooms for high-volume centers • Setup and turnover efficiency
	Teaching capabilities	Variable	• Dual-console availability for academic programs • Telestration and guidance features • Ability to control trainee actions
	Instrument reach	High	• Access to upper abdomen for comprehensive staging • Pelvic sidewall access for lymph node dissection • Ability to perform hysterectomy and oophorectomy without repositioning
Thoracic surgery	Port placement	Critical	• Arm configuration for limited intercostal access • Minimal external arm collisions • Capability for single-lung ventilation cases
	Instrument length	Critical	• Reach to posterior mediastinum • Access to superior sulcus • Capability for subxiphoid approach if utilized
	Stapling integration	High	• Compatibility with thoracic staplers • Angulation capabilities for bronchial closure • Surgeon control of stapling devices
	Visualization	Critical	• Image quality for hilar dissection • Illumination of posterior recesses • Identification of segmental anatomy
	Setup efficiency	High	• Rapid deployment for physiologically challenging cases • Reproducible positioning • Minimal room reconfiguration requirements
Cardiac surgery	Instrument reach and length	Critical	• Extended reach for intracardiac procedures • Access to all cardiac chambers • Capability for minimally invasive approaches (mini-thoracotomy)
	Stability and precision	Critical	• Minimal tremor for coronary anastomoses • Stable platform for beating heart procedures • Precise control for valve repair
	Specialized instruments	High	• Compatibility with cardiac-specific tools • Microforceps for delicate tissues • Integration with stabilization devices
	Visualization	High	• High-definition imaging for small vessels • Adequate field of view in confined spaces • Integration with cardiac imaging (TEE)
	Setup efficiency	Medium	• Rapid deployment for urgent cases • Streamlined positioning for lateral access • Efficient instrument exchange |
ENT/Head and neck surgery	Compact design	Critical	• Small profile arms for transoral access • Minimal external collision in confined spaces • Flexible camera positioning
	Visualization	Critical	• Angled scopes for transoral approaches • High magnification for microstructures • Excellent illumination in deep cavities
	Specialized instruments	High	• Fine-tipped instruments for delicate dissection • Compatible with nerve monitoring • Appropriate for mucosal work
	Tremor filtration	High	• Enhanced stability for microsurgery • Precise control near critical structures • Smooth motion scaling
	Integration capabilities	Medium	• Compatibility with navigation systems • Nerve monitoring integration • Coordination with imaging
Pediatric surgery	Instrument scaling	Critical	• Appropriately sized instruments for small patients • Reduced instrument diameter • Pediatric-specific tool availability
	Motion scaling	Critical	• Enhanced precision in small spaces • Greater motion scaling ratios • Fine control for delicate tissues
	Visualization	High	• Adequate depth perception in small cavities • Appropriate camera size for pediatric spaces • High magnification capabilities
	Versatility	High	• Adaptability across age ranges • Multi-specialty capability (urology, general, thoracic) • Quick reconfiguration between cases
	Safety features	High	• Enhanced haptic feedback for tissue handling • Collision avoidance in small spaces • Careful energy device integration

#### General surgery

Requires exceptional procedural diversity and platform versatility, accommodating a case mix ranging from cholecystectomies to complex hepatobiliary resections. Economics presents challenges as many common procedures have narrow contribution margins. Training requires a comprehensive curriculum addressing this procedural spectrum [26].

#### Urology

Prioritizes visualization capabilities, instrument dexterity, and multi-arm functionality, particularly for prostatectomy and partial nephrectomy. Advanced visualization technologies like near-infrared fluorescence imaging provide significant advantages for identifying vascular anatomy and assessing renal perfusion.

#### Gynecology

Spans straightforward procedures like benign hysterectomy to complex operations such as lymph node dissection and fertility-sparing myomectomy. The shift toward outpatient procedures influences platform selection, favoring systems with smaller footprints and rapid setup times.

#### Thoracic surgery

Requires systems functioning effectively with limited port placement options and fixed anatomical boundaries. Single-lung ventilation creates time constraints, making platform setup efficiency particularly important.

#### Cardiac surgery

Demands specialized features including extended instrument reach, exceptional stability for anastomoses, and integration with cardiac-specific technologies. The unforgiving nature of cardiac tissues requires platforms with superior precision and minimal latency.

#### ENT/Head and neck surgery

Benefits from compact platforms with enhanced visualization for confined spaces. Transoral robotic surgery requires specialized instrumentation and camera angulation, while preserving critical neurovascular structures.

#### Pediatric surgery

Presents unique challenges with smaller working spaces and delicate tissues. Platforms must accommodate instrument scaling, provide exceptional haptic sensitivity, and allow precise control in miniaturized surgical fields. Case volumes often limit dedicated pediatric robotic programs to specialized centers.

### Specialty-focused features assessment

Institutions should evaluate platforms based on their highest volume and highest-priority procedures, as platform capabilities vary significantly across surgical applications. [32] This assessment requires examining both published evidence and practical performance characteristics.

### Platform architecture and procedural fit

Different robotic architectures offer distinct advantages for specific procedures and institutional needs. [19] (see Technical Assessment for comprehensive platform comparison).

### Evidence-based platform evaluation

When evaluating platforms, institutions must consider the evidence maturity for each surgical application. Established systems like da Vinci have achieved IDEAL Stages 3–4 for procedures like prostatectomy and hysterectomy. [18] In contrast, newer platforms currently lie in Stages 1–2, indicating limited clinical experience and potentially greater implementation risks.

### Procedural complexity matching

Platform capabilities must align with institutional case complexity. Key considerations include:

- Navigation of anatomically confined spaces (pelvis, mediastinum, pediatric abdomen)
- Visualization performance in challenging regions requiring careful assessment of the field of view, illumination, and image quality
- Precision requirements for microsurgical or reconstructive procedures, where tremor filtration and motion scaling differences directly impact outcomes [33].

### Multi-specialty utilization

Maximizing ROI typically requires cross-specialty platform utilization. Research demonstrates variable skill transferability between platforms, with some systems showing shorter learning curves for surgeons with prior robotic experience. [34] Efficient instrument changeover and minimal reconfiguration time between specialties significantly impact OR utilization rates and financial performance [27].

## Technical assessment of robotic platforms

### Core system architecture

Fundamental architectural differences between robotic platforms significantly impact their integration into surgical workflows. Open-console versus closed-console designs should be evaluated based on systematic surgeon preference assessments. Modular versus integrated system approaches offer different advantages for institutional flexibility and space utilization. Camera control mechanisms, such as eye-tracking (as in the Senhance system), hand controls (da Vinci), or voice-activated systems, affect workflow efficiency and surgeon learning curves differently across specialties.

A structured evaluation using the STARSS tool can help quantify these differences. [34] This evaluation considers surgeon preferences, cognitive load, and task performance. The STARSS tool developed by SAGES [35] is another mechanism for assessing robotic platforms, comparing software, hardware, usability, safety, ergonomics, and training.

### Instrument and visualization features

The technical capabilities of robotic instruments represent a primary differentiator between platforms. Degrees of freedom in instrument articulation range from simple wrist-like movements to complex multi-axial articulation systems. Haptic feedback systems vary significantly between platforms, with potential implications for surgical safety and learning curves. The levels of haptic sophistication range from basic force feedback to more advanced tissue differentiation capabilities, with possible implications for surgical applications requiring delicate tissue manipulation [10].

Visualization systems differ in field of view dimensions (manufacturer specifications typically range from 70° to 110°), magnification capabilities (generally 10–15 × in high-end systems), and advanced imaging overlays. The Senhance system employs a unique eye-tracking camera control system, while platforms like AVATERA and da Vinci use different approaches to visualization. Advanced image overlay features, such as fluorescence imaging (available on da Vinci systems with Firefly technology) and integration of preoperative imaging data, provide significant advantages for oncological and vascular procedures [11].

### Ergonomics and user experience

Surgeon comfort and system usability significantly impact adoption, utilization, and sustainability of a robotic program. Comprehensive evaluation should include surgeon-led assessments of console ergonomics, hand controller designs, physical space considerations, and user interface.

User interface evaluation should include standardized task performance metrics, cognitive load assessment, and surgeon preference scales to quantify differences between platforms [34].

## Analysis of institutional integration

### Infrastructure requirements

The physical integration of robotic systems into existing facilities requires careful evaluation of infrastructure compatibility. Floor loading requirements, ranging from 650–1200 kg depending on platform, prove critical particularly in older buildings or upper floors. [22, 25] Electrical requirements vary from standard 120 V circuits for smaller systems to dedicated 240 V power with uninterruptible backup for larger platforms.

Operating room dimensions must accommodate substantial space requirements for robotic platforms, with additional clearances needed for optimal team movement and equipment positioning [25, 36].

### Existing technology integration

Compatibility with current institutional technologies significantly impacts the total cost and effectiveness of robotic platform integration. Operating room table systems must interface effectively with the robotic platform, with some systems requiring specialized tables representing substantial additional capital investments for robotic-compatible models with advanced positioning capabilities.

Integration capabilities extend beyond physical compatibility to include data systems. When platforms involve data exchange with vendors or cloud-based analytics, institutions must ensure appropriate cybersecurity measures and liability management. Critical considerations include compliance with data privacy regulations, secure transmission protocols, and clear delineation of data ownership and breach responsibilities. Export capabilities for integration with institutional electronic medical records and research databases vary significantly between platforms, affecting both clinical workflow and research capabilities [19].

### Support and training infrastructure

The quality and accessibility of training programs significantly impact successful platform adoption. Vendor training curricula should be evaluated against the specific learning needs of the institution’s surgical staff, considering both initial certification and ongoing skill development.

Current credentialing requirements for robotic surgery vary widely, with significant differences in simulation assessment, case observation requirements, and proctoring standards. Institutions should evaluate whether platform-specific training programs meet appropriate standards for ensuring surgeon proficiency, with attention to validated assessment methods and progression criteria. [28] Onsite versus remote support capabilities during cases, and ensuring vendors can meet the complex needs of multi-specialty robotic teams, are additional important considerations when evaluating platform support infrastructure.

## Educational infrastructure and training pathways

### Simulation capabilities and pre-clinical training

Educational excellence begins with robust simulation resources that allow surgeons and trainees to develop skills before patient contact. Platforms differ significantly in their simulation offerings. Institutions should evaluate whether simulation systems offer graduated difficulty levels, objective performance metrics, and validated curricula that align with established surgical education principles. [26, 37] Studies demonstrate that robotic surgical simulation, combined with structured training programs and proctorship, can accelerate skill acquisition, with research showing that novice robotic surgeons who trained on virtual simulators outperformed control groups with no simulator exposure. However, systematic reviews reveal that the impact of simulation training on learning curves remains poorly quantified, with only 8% of studies providing data on pre-clinical training. [38] The availability of mobile or portable simulation units can significantly enhance accessibility for residents and fellows, maximizing training opportunities outside of clinical hours.

### Clinical teaching infrastructure

Dual-console systems allow mentors to actively participate in procedures, while telestration features enable instructors to highlight anatomical structures without interrupting procedures. Some platforms offer additional educational tools such as video recording with annotation capabilities, case libraries for review, and integration with learning management systems for tracking educational progress [28].

### Proficiency-based progression frameworks

Structured educational pathways ensure competency before independent practice. Institutions should evaluate whether platforms offer standardized assessment tools, performance dashboards, and integration with credentialing processes. The ability to track individual surgeon and trainee progress through objective metrics facilitates quality assurance and helps identify areas requiring additional training, with validated assessment tools like GEARS demonstrating strong correlation between simulation performance and operative outcomes [26, 39].

## Assessment of vendor relationship

### Company stability and vision

The long-term viability of a robotic platform depends significantly on the stability and strategic direction of its manufacturer. Established systems offer stability but at premium pricing. In contrast, smaller robotics companies offer innovative approaches but with less established financial histories, with market analysis showing that vendor financial stability directly correlates with long-term platform support and upgrade availability. [22, 40] The size and growth trajectory of each platform’s installed base affect everything from peer learning opportunities to the stability of instrument supply chains, with studies demonstrating that platforms with smaller installed bases face greater risks of supply chain disruptions and reduced vendor support [22].

### Support services comparison

The quality of clinical and technical support directly impacts program success and sustainability. Clinical specialist availability and expertise vary considerably between vendors, affecting the level of in-room support available during initial implementation and for complex cases. Technical service response times and resolution rates should be verified through discussions with existing customers rather than relying solely on contractual guarantees.

### Contract flexibility

Negotiated terms for future system evolution and technology upgrade significantly impact long-term satisfaction and cost-effectiveness. Terms for additional system acquisition become particularly important for growing programs that may require expansion beyond a single robot. Warranty coverage and exclusions should be thoroughly analyzed, with particular attention to how user error is defined and what remedies are available for system downtime. [22] Contract negotiations should also address liability allocation, particularly regarding cybersecurity breaches, data protection, and insurance requirements. It is the buyer’s responsibility to ensure the vendor can meet these requirements.

## Economic evaluation and financial models

### Acquisition cost structures

The financial model for acquiring robotic technology extends far beyond the headline purchase price. Institutions should conduct detailed comparisons of capital expenditure requirements across platforms, including accessories, instruments, and facility modifications. Lease options and payment structures vary significantly between vendors. Usage-based arrangements and risk-sharing models where payment is partially tied to case volume are now available, and programs for trading in existing robotic equipment can substantially offset initial costs for institutions upgrading from older systems [27].

### Operational cost analysis

Ongoing operational expenses often exceed initial acquisition costs over the lifetime of a robotic platform. Per-case costs include both disposable components and reusable instruments with limited lifespans, with studies showing disposable instrument costs ranging from $1,500–$3,500 per case depending on procedure complexity. The number of uses of instruments is therefore important. [14, 17] Annual service contract terms vary substantially, with significant differences in standard versus premium coverage, typically representing 8–12% of initial acquisition costs annually with premium service contracts including faster response times and enhanced training support [14].

### Total cost of ownership modeling

Comprehensive economic evaluation requires detailed 5-year cost projections for each platform option based on case volume projections and reimbursement models relevant to the institution’s payer mix.

Future upgrade pathways and compatibility costs represent a significant component of long-term expenditure. Technology longevity assessment should include evaluation of each vendor’s historical patterns of platform support and the competitive landscape of robotic surgery innovation.

When comparing total cost of ownership between platforms, the analysis should include all direct costs (acquisition, per-case expenses, maintenance) and indirect financial impacts (operating room efficiency, length of stay reductions, complication rate differences). Platform selection decisions should be based on comprehensive financial modeling rather than focusing primarily on acquisition price [36].

### Financial Models: academic, community, and specialty center projections

The economic implications of robotic platform selection vary significantly based on institutional characteristics, case mix, and implementation approach. The following scenarios, derived primarily from experience with established platforms, especially Intuitive Surgical platforms, provide general guidance, though specific outcomes may vary with newer systems offering different pricing models and acquisition costs.

### Scenario 1: academic medical center with multi-specialty program

Academic hospitals implementing multi-specialty robotic programs typically experience extended periods before profitability, with traditional purchase models showing negative contribution margins during the first 24–30 months. [25] Academic medical centers typically require substantial case volumes for financial sustainability. Research demonstrates clear associations between higher surgical volumes and improved financial performance. [41] However, precise breakeven thresholds remain poorly defined in the literature and require institution-specific financial modeling. Established academic programs show profitability at volumes ranging from 586 to over 600 cases annually [27].

Key value drivers include commercial insurance contribution margins, reduced length of stay (mean reduction 0.23 days compared to laparoscopic surgery and 1.69 days compared to open surgery depending on procedure), [4, 42] decreased readmissions in specific procedures, for example, studies showing significant reductions in 30-day readmission rates for rectal cancer surgery, [43] and enhanced referrals. The integration of robotic technology with residency and fellowship training programs can enhance recruitment and create additional revenue streams through educational grants and research funding.

### Scenario 2: community hospital with focused service line

Community hospitals implementing targeted robotic service lines face variable financial trajectories depending on platform selection and financing approach. With facilities typically requiring 18–36 months to breakeven based on industry experience, though newer platforms with lower acquisition costs or per-case pricing may accelerate this timeline. Community hospitals face variable financial sustainability timelines, with rural critical access hospitals averaging approximately 106 robotic procedures annually, [44] though profitability appears closely linked to achieving higher surgical volumes and favorable payer mix.

The primary value drivers are market differentiation, surgeon recruitment/retention, reduced patient transfers to tertiary centers, and decreased complication rates [36].

### Scenario 3: specialty surgical center with focused utilization

Specialty surgical centers focusing on high-margin procedures demonstrate the most favorable economics, and positive returns are possible within 12–18 months depending on platform costs and pricing model. Financial outcomes remain highly sensitive to commercial payer mix, with centers maintaining > 70% commercial insurance typically achieving positive margins at volume thresholds that vary by platforms [27, 45].

These centers benefit from high contribution margins from commercially insured specialty cases, focused team efficiency through case consistency, with studies demonstrating that system setup time decreases as operating team experience increases, [46] and marketing advantages in competitive specialty environments.

While specific volume thresholds vary based on institutional characteristics, research demonstrates clear associations between higher surgical volumes and improved financial performance in robotic surgery programs. [41] However, precise breakeven thresholds remain poorly defined in the literature and require institution-specific financial modeling incorporating local factors including payer mix, case complexity, acquisition costs, and operational efficiency.

### Implementation recommendations based on economic scenarios

These scenarios suggest several principles for optimizing economic outcomes:

1. *Prioritize favorable reimbursement procedures*: Begin with procedures showing the strongest financial profiles while teams build efficiency. Analysis of robotic program implementation demonstrates this approach can accelerate time to breakeven by 6–12 months compared to more diverse initial case selection [27].
2. *Recognize minimum volume thresholds*: Research consistently identifies minimum annual case volumes required for financial sustainability, though these vary by platform and pricing model. As detailed in Financial Models, minimum case volume requirements vary significantly by institutional type and platform selection. Programs failing to achieve appropriate thresholds typically show persistent negative financial performance [36].
3. *Incorporate realistic efficiency curves*: Learning curve requirements vary significantly by procedure complexity and surgeon experience. Learning curve requirements vary significantly by procedure complexity and surgeon experience. A systematic review found substantial variation, with 12–140 cases needed for urological procedures, 0–80 for general surgery, and 0–74 for colorectal procedures, though the authors noted that learning curve estimates were subject to considerable uncertainty due to limitations in study design and heterogeneous methods, [47] while simpler procedures may require as few as 12–15 cases for operative efficiency.
4. *Consider alternative acquisition models*: Financial analysis demonstrates that different acquisition approaches (purchase, lease, per-case pricing) significantly impact financial performance based on projected volume and capital constraints. Per-case pricing models typically become less economical above 200–250 annual cases, while purchase models show superior long-term economics for high-volume programs. However, these thresholds shift with varying platform costs and contract terms [48].
5. *Include indirect benefits in economic assessment*: Comprehensive financial modeling should incorporate measurable indirect benefits including reduced complications (31% lower risk of severe complications in rectal surgery, with overall complication rates varying by procedure), [29] shorter lengths of stay (mean reduction 0.23–1.69 days depending on comparison procedure), [4] and advantages in physician recruitment and market positioning [12].

## Implementing a structured selection process: tools and stakeholder engagement

Healthcare institutions require formal evaluation methodologies for robotic platform selection. Research identifies a validated hierarchy of decision criteria: clinical outcomes and technical capabilities rank highest, economic considerations intermediate, and vendor reputation lower. [21] Multi-Criteria Decision Analysis (MCDA) integrates quantitative and qualitative factors into comprehensive assessment [13].

Side-by-side comparative trials using standardized surgical tasks provide objective performance data, including both simple tasks and complex procedure-specific simulations approximating real surgical challenges [34].

### Stakeholder engagement

Systematic integration of stakeholder perspectives is essential:

- *Surgeons*: Structured feedback after extended evaluation periods using validated assessment tools measuring ergonomics, cognitive workload, and usability. [14] This is important as surgeons should eventually be accountable for operating room time and case selection, with metrics that address procedure duration, complications, and other outcome measures.
- *Educational leaders*: Program directors and education committees evaluate platforms against training objectives and future needs [26].
- *Operating room teams*: Nursing and technical staff assess workspace configuration, instrument management, and efficiency. Staffing considerations are particularly important given nursing shortages, and some level of case prioritization may be required when access becomes an issue.
- *Administration*: Alignment with strategic priorities and fiscal constraints, considering opportunity costs and genuine patient needs versus market positioning [45].

### Decision support tools

**Fig. 1:**
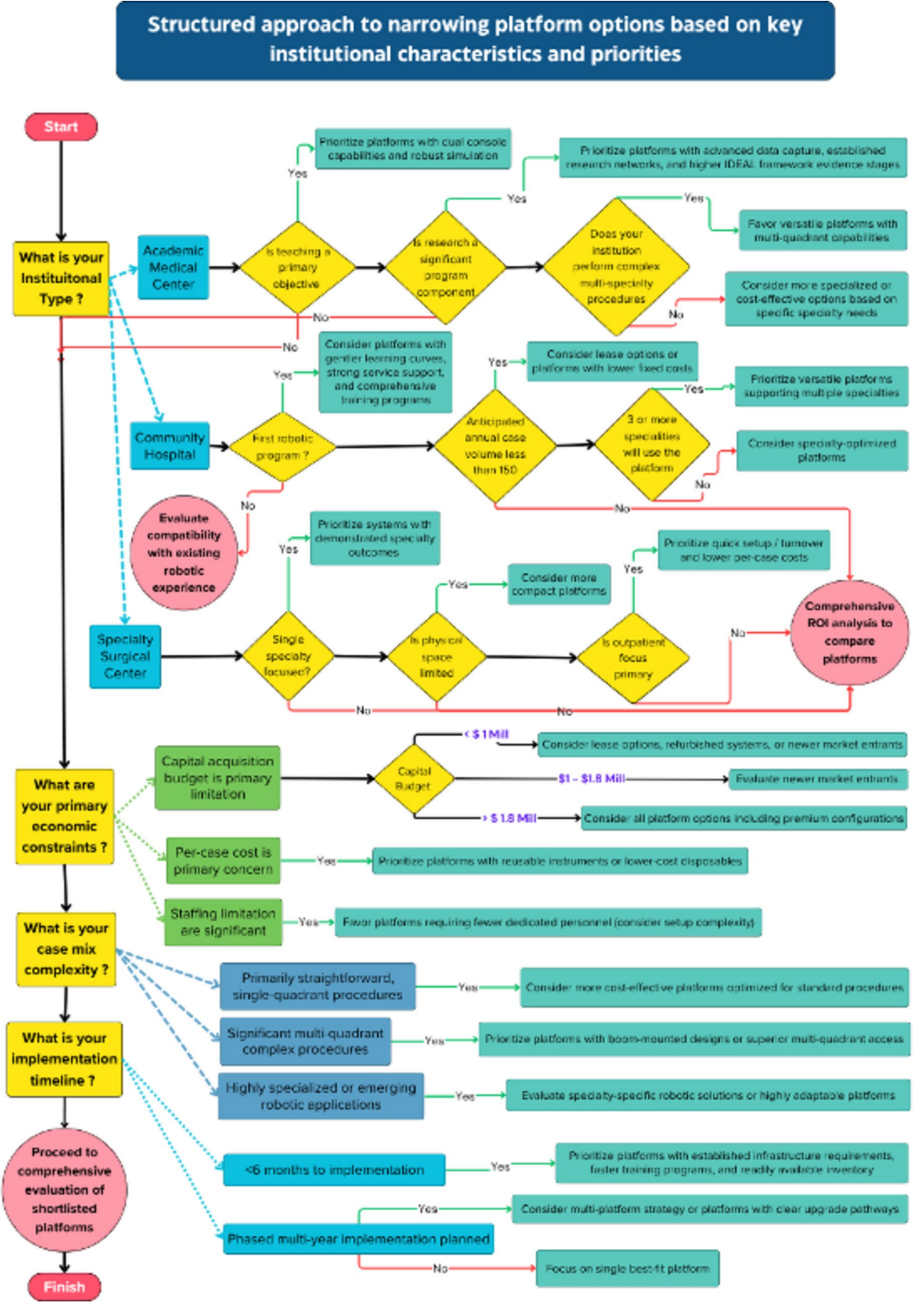
Decision tree for initial platform screening based on institutional type and priorities

A sequential approach proves most effective:

- Preliminary decision tree Fig. 1 narrows options based on critical requirements.
- Weighted decision matrix Table 2 with explicit institutional priorities.
- Sensitivity analysis identifies robust conclusions and where additional information is needed [13].

**Table 2:** Customizable weighted decision matrix template for platform evaluation

Evaluation domain with weight	Criterion	Platform A score (1–5)	Platform A weighted	Platform B score (1–5)	Platform B weighted
Procedure-specific evaluation (Weight 25%)	Suitability for primary procedures				
	Evidence quality for key procedures				
	Instrument availability for specialties				
	Multi-quadrant access capabilities				
	Specialty-specific advantages				
Technical capabilities (Weight 20%)	Console design and ergonomics				
	Instrument articulation and dexterity				
	Visualization system quality				
	Energy device integration				
	Haptic feedback capabilities				
Institutional compatibility (Weight 15%)	Space and infrastructure requirements				
	Integration with existing equipment				
	Ease of setup and turnover				
	Data integration capabilities				
	Impact on workflow efficiency				
Economic considerations (Weight 20%)	Initial acquisition cost				
	Per-case operational costs				
	Service and maintenance expenses				
	Projected return on investment				
	Upgrade pathway costs				
Vendor relationship (Weight 10%)	Company stability and market position				
	Service responsiveness and quality				
	Training program comprehensiveness				
	Contract flexibility and terms				
	Innovation pipeline and roadmap				
Implementation factors (Weight 10%)	Ease of implementation				
	Learning curve considerations				
	Training resources available				
	Timeline to operational efficiency				
	Change management requirements				
TOTAL SCORE					

The following matrix template can be customized to reflect an institution’s specific priorities by adjusting the weights. Each platform under consideration should be scored on a scale of 1–5 for each criterion, with the weighted scores calculated by multiplying the raw score by the assigned weight

**Application guidelines**

**Customize weights based on institutional priorities:** Academic centers may increase weight on teaching capabilities and research potential, while community hospitals might emphasize economic factors and ease of implementation

**Involve diverse stakeholders in scoring:** Each stakeholder group should independently score the platforms to capture different perspectives before aggregating results

**Conduct sensitivity analysis:** Adjust weights to determine if small changes significantly affect outcomes, which may indicate areas requiring further evaluation

**Document rationale for scores:** Beyond numerical ratings, maintain detailed notes explaining the reasoning behind each score to facilitate discussion and decision-making

**Revisit scoring after site visits and hands-on evaluation:** Initial scores based on specifications and vendor presentations should be updated after direct experience with each platform

## Emerging technologies and future considerations

As robotic surgery technology rapidly evolves, institutions must consider future technological trajectories alongside current capabilities to ensure today’s investments remain aligned with tomorrow’s surgical innovations.

### Environmental sustainability

Environmental impact is becoming an increasingly important consideration for healthcare institutions evaluating robotic platforms. Studies have shown that robotic procedures can result in 43.5 percent higher greenhouse gas emissions and 24 percent higher waste production than laparoscopic alternatives. [30] Institutions should evaluate platforms based on energy efficiency, reusable versus disposable instrument options, and the manufacturer’s commitment to sustainable practices. As regulations tighten—evidenced by initiatives like The Joint Commission’s certification in sustainable healthcare [49]—platforms offering reusable devices with extended lifecycles, reduced energy requirements, and options for instrument reprocessing will likely offer advantages in meeting institutional sustainability goals.

### Artificial intelligence integration

AI capabilities vary significantly across platforms, though specific applications remain in development. While da Vinci systems gradually incorporate machine learning through Intuitive’s digital ecosystem, the practical end-user applications are still evolving. Medtronic positions Hugo RAS as explicitly AI-ready. However, implementation of AI features in surgical robotics remains in early development stages across all platforms. [20, 50] Asensus Surgical’s Senhance platform has introduced Intelligent Surgical Unit™ technology purportedly providing real-time intraoperative guidance through machine vision. Evaluation should consider vendors’ AI development roadmaps, including surgical workflow optimization, anatomical structure recognition, and predictive analytics for decision support. Platforms with established FDA clearance processes may deploy new AI features more rapidly [51].

### Augmented reality and advanced visualization

Next-generation visualization technologies will transform robotic surgery through enhanced intraoperative guidance. Platforms differ in integrating preoperative imaging (CT, MRI, ultrasound) with real-time surgical visualization. The Senhance system demonstrates early capabilities, while others develop features for highlighting critical structures, surgical planes, and resection margins. Advanced spectral imaging for real-time tissue classification and perfusion assessment is emerging. These capabilities are particularly valuable for complex oncological procedures and surgeries involving critical neurovascular structures [33].

### Remote surgery and telementoring

While regulatory and connectivity challenges limit the implementation of telesurgery, telementoring capabilities are immediately relevant. Platforms vary in supporting telestration, dual-console control sharing, and integrated communication systems. Da Vinci’s dual-console configuration has established telementoring capabilities, while newer systems develop alternative approaches to collaborative surgery [32].

### Miniaturization and specialty-specific robotics

The trend toward procedure-specific systems is accelerating. Emerging miniaturized platforms—such as Johnson & Johnson’s Monarch Platform for bronchoscopic procedures and MIRA miniature robot—suggest diversified robotic technologies. Institutions should evaluate whether current investments position them to adopt specialized systems as complements to general-purpose platforms and assess integration potential. [19, 20].

### Data integration and interoperability

Platforms vary significantly in capturing procedural data, integrating with institutional data warehouses, and supporting interoperability. These capabilities become increasingly important as surgical data gain value [22].

When evaluating platforms, institutions should discuss vendors’ development roadmaps, upgrade pathways, and strategies for incorporating emerging technologies. The capacity for technological evolution without platform replacement is critical for maximizing long-term investment value [18].

## Ten critical pitfalls in platform selection and mitigation strategies

Understanding common mistakes in robotic platform selection can help institutions navigate this significant strategic decision more effectively.

1. *Overemphasis on acquisition cost and incomplete financial modeling*—Focus on total cost of ownership over 5–7 years through comprehensive financial models that include maintenance, instruments, training, upgrades, and operational expenses. Create customized projections for your specific institutional environment with sensitivity analyses for key variables. Verify all costs with existing users rather than relying solely on vendor estimates. Platform selection decisions should be based on complete financial modeling rather than focusing primarily on acquisition price [27].
2. *Inadequate surgeon involvement*—Establish selection committees with balanced representation from surgical specialists, nursing, administration, and finance. Use structured evaluation tools quantifying surgeon preferences, requiring hands-on evaluation by all anticipated users [45].
3. *Failure to consider procedural applicability*—Develop detailed profiles of anticipated robotic case mix, including anatomical considerations and procedure-specific needs. Evaluate platform performance for highest volume procedures through literature review and institutional consultation [32].
4. *Underestimating infrastructure requirements*—Conduct detailed site assessments with engineering involvement before selection. Request precise specifications including footprint, clearances, and infrastructure needs. Visit similar institutions to observe systems in comparable environments [25].
5. *Neglecting learning curve impact*—Account for known learning curve patterns. Develop realistic timeline projections and consider phased implementation during lower-volume periods [28].
6. *Inadequate educational infrastructure assessment*—Evaluate simulation fidelity, dual-console availability, objective assessment tools, and curriculum integration. Consider long-term impact on training programs. [26] Include comprehensive perioperative team education, not just surgeon training. Designate “super users” for advanced support during implementation [34].
7. *Insufficient due diligence on vendor stability*—Review financial statements, funding sources, and market assessments. For private companies, request evidence of sufficient capitalization. Consider financial performance guarantees in contracts [19].
8. *Ignoring integration with existing technology*—Catalog all interfacing technologies and explicitly verify compatibility. Request detailed integration specifications and test in simulated environments when possible [22].
9. *Neglecting long-term technological evolution*—Evaluate vendor innovation pipelines, upgrade pathways, and backward compatibility patterns. Include contractual provisions for future developments and predictable upgrade pricing [20].
10. *Overlooking liability and risk management*—Cyber risk and liability allocation are frequently underestimated in platform selection. When platforms involve data exchange or cloud-based analytics, ensure vendors maintain adequate cyber liability and general liability insurance structured to survive vendor bankruptcy or acquisition. With healthcare cybersecurity incidents increasing—92% of healthcare providers reported at least one cyberattack in 2024 [52]—and robotic systems representing high-value targets for cyber attacks, [52] contract terms must clearly delineate responsibilities for data breaches, system failures, and patient harm. Explicit attention to liability management during contract negotiation prevents significant institutional exposure, particularly given that medical device cybersecurity breaches can result in average costs exceeding $7 million per incident [53, 54].

By proactively addressing these pitfalls, institutions can improve their likelihood of selecting the optimal robotic platform for their specific needs [18].

## Discussion

The comprehensive framework presented addresses a critical gap in robotic surgery platform evaluation at a pivotal time in the field’s evolution. As the robotic surgery marketplace expands beyond its historical monopoly, institutions face increasingly complex decisions extending beyond technical specifications to encompass strategic, educational, and economic considerations. This eight-domain framework provides the systematic approach necessary for navigating this complexity while ensuring decisions align with institutional missions and capabilities.

This framework differs substantially from existing evaluation tools in both scope and intent. The SAGES Tool for Assessing Robotic Surgery Systems (STARSS) tool provides detailed technical metrics comparing software, hardware, usability, safety, ergonomics, and training features across platforms. [35] While STARSS excels at objective technical comparison, this framework extends evaluation to institutional strategy, implementation planning, and long-term sustainability. The tools are complementary—STARSS addresses technical capabilities while this framework determines which platform best serves institutional needs. This distinction proves critical as technically superior platforms may be suboptimal for institutions lacking appropriate infrastructure, case volume, or financial resources.

The framework’s strength lies in comprehensive coverage and adaptability. Integration of established methodologies (IDEAL Framework and MCDA) provides evidence-based structure while maintaining flexibility for institutional customization. The eight domains ensure all stakeholder perspectives receive consideration, from surgeon preferences to administrative constraints. Practical tools—decision tree for initial screening, weighted matrix for detailed comparison, and implementation checklist—transform theoretical concepts into actionable evaluation processes.

Several limitations require acknowledgment. Framework development relied on expert opinion and literature review rather than formal consensus methodology such as a Delphi process. Economic projections derive primarily from established platform data, potentially limiting applicability to newer systems with alternative pricing models. The framework requires institutional commitment to customize weightings and priorities. Additionally, rapid technological advancement necessitates regular updates to maintain relevance.

Future development should address these limitations while expanding utility. Prospective validation studies comparing selection outcomes between systematic versus ad hoc approaches would strengthen the evidence base. Digital decision-support tools could streamline application while ensuring consistent evaluation. Integration with value-based healthcare metrics would align platform selection with broader transformation initiatives. As surgical robotics evolves toward greater autonomy, the framework must adapt to evaluate emerging capabilities.

## Conclusion

The selection of robotic surgery platforms represents one of the most consequential decisions facing modern surgical departments, with implications for clinical outcomes, educational missions, and institutional finances. The framework in this paper provides the systematic approach essential for navigating an increasingly complex marketplace. The eight key principles—evidence-based clinical evaluation, institutional compatibility assessment, technological capability analysis, meaningful surgeon engagement, robust educational infrastructure, thorough economic modeling, regulatory compliance consideration, and future sustainability planning—provide guideposts for successful implementation. By adopting this evidence-based approach, institutions can move beyond marketing claims to make decisions grounded in objective evaluation. As robotic surgery continues toward becoming the standard of care, institutions that thrive will be those selecting platforms not just for current needs but with vision for future possibilities. Healthcare leaders are encouraged to embrace this systematic methodology, recognizing that rigorous evaluation leads to better decisions, successful implementations, and ultimately, improved patient care.

## Supplementary Information

Below is the link to the electronic supplementary material.

Supplementary file1 (DOCX 18 kb)
