# Supplementary material for: White paper on effective preacquisition evaluation of soft tissue robotic surgery platforms for healthcare institutions
Source: Surg Endosc. 2026 Feb 24;40(3):1797–814. doi: 10.1007/s00464-026-12637-4 (PMC12971938; doi:10.1007/s00464-026-12637-4)
Supplement: Supplementary file 1 — Supplementary file1 (DOCX 18 kb) [file 464_2026_12637_MOESM1_ESM.docx]

**Appendix A: Implementation Checklist for Comprehensive Platform Selection**

The following structured checklist provides a practical tool for institutions to ensure comprehensive consideration of all relevant factors in the robotic platform selection process.

**I. Preliminary Assessment**

- Established multidisciplinary selection committee with representation from all stakeholders
- Completed current and projected case volume analysis by specialty and procedure type
- Identified strategic objectives for robotic program (clinical, financial, research, education)
- Assessed existing infrastructure and potential constraints (space, electrical, structural)
- Established budget parameters and financial evaluation criteria
- Determined timeline requirements for implementation

**II. Procedure-Specific Evaluation**

- Documented procedure types representing 80% of anticipated robotic case volume
- Identified anatomical access requirements (single quadrant vs. multi-quadrant)
- Assessed specialties' specific instrumentation requirements
- Reviewed published outcomes data for primary procedures on each platform
- Evaluated IDEAL framework stage of evidence for each platform by specialty
- Discussed specific procedural requirements with surgeons from each specialty

**III. Technical Capabilities Assessment**

- Compared core architecture (open vs. closed console, fixed vs. mobile platform)
- Evaluated instrument capabilities (articulation, energy devices, specialized tools)
- Assessed visualization systems (image quality, field of view, special imaging modes)
- Examined ergonomics and user interface from surgeon perspective
- Compared setup and turnover processes from nursing perspective
- Evaluated noise levels, heat generation, and environmental impact

**IV. Institutional Compatibility**

- Verified OR space requirements and compatibility with existing room configurations
- Assessed floor loading requirements relative to facility capabilities
- Evaluated electrical and networking infrastructure requirements
- Confirmed compatibility with existing OR tables and positioning equipment
- Determined integration capabilities with existing imaging and navigation systems
- Assessed compatibility with electronic medical record systems for data capture

**V. Support and Training Infrastructure**

- Compared vendor training programs for surgeons and OR staff
- Evaluated simulation resources and capabilities
- Assessed availability of proctoring support during initial implementation
- Compared technical support response times and availability by region
- Determined requirements for biomedical engineering support and training
- Evaluated resources for troubleshooting and ongoing education

**VI. Economic Analysis**

- Compared acquisition options (purchase, lease, per-case pricing models)
- Calculated 5-year total cost of ownership projections
- Determined per-case costs for typical procedures in your institution
- Assessed maintenance and service contract terms and costs
- Evaluated instrument lifecycle and replacement costs
- Projected revenue impacts based on case volume, payer mix, and reimbursement

**VII. Vendor Assessment**

- Researched vendor financial stability and market position
- Evaluated installed base size and geographic distribution
- Assessed innovation pipeline and future technology roadmap
- Reviewed customer satisfaction data from similar institutions
- Evaluated contract terms for flexibility, upgrades, and service guarantees

**VIII. Implementation Planning**

- Determined physical infrastructure modifications required
- Developed training plan for initial surgical team
- Established credentialing criteria for robotic surgeons
- Created timeline for program expansion to additional surgeons and specialties
- Developed metrics for program evaluation and continuous improvement
